# Adherence to the Mediterranean Diet and Inflammatory Markers

**DOI:** 10.3390/nu10010062

**Published:** 2018-01-10

**Authors:** Antoni Sureda, Maria del Mar Bibiloni, Alicia Julibert, Cristina Bouzas, Emma Argelich, Isabel Llompart, Antoni Pons, Josep A. Tur

**Affiliations:** 1 Research Group on Community Nutrition and Oxidative Stress, University of Balearic Islands, E-07122 Palma de Mallorca, Spain; tosugo@hotmail.com (A.S.); mar.bibiloni@uib.es (M.d.M.B.); aliciajulibert@gmail.com (A.J.); cristinabouvel@gmail.com (C.B.); eargelich15@gmail.com (E.A.); isabel.llompart@ssib.es (I.L.); antonipons@uib.es (A.P.); 2 CIBER Fisiopatología de la Obesidad la Nutrición (CIBEROBN)-Instituto de Salud Carlos III, E-07122 Palma de Mallorca, Spain

**Keywords:** adiponectin, Balearic Islands, cytokine, dietary questionnaire, leptin, Mediterranean diet

## Abstract

The aim was to assess inflammatory markers among adults and adolescents in relation to the adherence to the Mediterranean diet. A random sample (219 males and 379 females) of the Balearic Islands population (12–65 years) was anthropometrically measured and provided a blood sample to determine biomarkers of inflammation. Dietary habits were assessed and the adherence to the Mediterranean dietary pattern calculated. The prevalence of metabolic syndrome increased with age in both sexes. The adherence to the Mediterranean diet in adolescent males was 51.3% and 45.7% in adults, whereas in females 53.1% and 44.3%, respectively. In males, higher adherence to the Mediterranean diet was associated with higher levels of adiponectin and lower levels of leptin, tumor necrosis factor alpha (TNF-α), plasminogen activator inhibitor 1 (PAI-1) and high-sensitivity C-reactive protein (hs-CRP) in adults, but not in young subjects. In females, higher adherence was associated with lower levels of leptin in the young group, PAI-1 in adults and hs-CRP in both groups. With increasing age in both sexes, metabolic syndrome increases, but the adherence to the Mediterranean diet decreases. Low adherence to the Mediterranean dietary pattern (MDP) is directly associated with a worse profile of plasmatic inflammation markers.

## 1. Introduction

The traditional Mediterranean diet (MD) is characterized by a high intake of vegetables, legumes, fruits and nuts and cereals (which in the past were largely unrefined), a high intake of olive oil, but a low intake of saturated lipids, a moderately high intake of fish (depending on the proximity of the sea), a low-to-moderate intake of dairy products (and then mostly in the form of cheese or yoghurt), a low intake of meat and poultry and a regular, but moderate intake of ethanol, primarily in the form of wine and generally during meals [1,2]. The traditional Balearic diet corresponds to the typical Mediterranean dietary pattern (MDP) [3]. Overall, the MDP is considered a healthy prudent dietary pattern, and a high adherence to it has been associated with a better health status, due to the protective effect that this pattern shows against various chronic diseases [4,5,6] including a favorable effect on total mortality, cardiovascular disease and several cancers [1,6,7]. The MD has been also proposed as one of the determinants of the longevity of these populations [8]. Adherence to a healthy dietary pattern has been shown to be inversely associated with metabolic syndrome (MetS) [9,10], some of its components [11] and type-2 diabetes [12]. The MetS is a cluster of common cardiovascular risk factors, including central obesity, hyperglycemia, low High-density lipoprotein (HDL)-cholesterol concentrations, hypertension and hypertriglyceridemia. The association of MetS with the risk of developing diabetes and cardiovascular disease (CVD) is well documented, implying a greater risk of mortality [13,14].

Lately, it has also been proposed that markers of systemic inflammation may be included in the MetS definition [15]. Although inflammation is a normal defense mechanism that protects the body against infection or other insults, pathological inflammation is associated with tissue damage and disease [16]. In this sense, low-grade systemic inflammation appears to play an important role in the pathophysiology of obesity, insulin resistance, CVD and the anomalous coagulation process [10,17]. The chronic inflammatory response is characterized by abnormal cytokine production and the activations of inflammatory signaling pathways [18,19]. Adipose tissue is currently considered to be hormonally active and to take part in the control of metabolism. Adipose tissue secretes a large number of physiologically-active peptides that have common properties with cytokines, and therefore referred to as adipocytokines [20]. Some of these adipocytokines are leptin, the inflammatory cytokines tumor necrosis factor α (TNF-α) and interleukin-6 (IL-6), plasminogen activator inhibitor 1 (PAI-1), resistin, angiotensinogen, as well as the anti-inflammatory cytokine adiponectin pro-inflammatory mediators and a reduced production of adiponectin [17,21,22]. This implies that measures focused on the reduction of the inflammatory situation would improve the risk factors associated with MetS. Studies assessing the association of the adherence to MD with diverse inflammatory biomarkers in healthy persons reported inverse correlations [23,24].

A growing body of evidence suggests that the protective effects of the MD may result, at least in part, from its anti-inflammatory properties [25]. Low-grade chronic inflammation has been involved as a potential mediator for the development of many cardio-metabolic diseases. Therefore, studying the relation between the diet quality and inflammatory markers will be interesting. The aim of this study was to assess the relationship between inflammatory markers and adherence to the MD in adults and adolescents of the Balearics Islands, a Mediterranean region, and to assess how the level of adherence to the MD could affect the levels of inflammatory markers.

## 2. Materials and Methods

### 2.1. Study Design

This study is part of two larger research projects in which the target population solely consisted of inhabitants aged between 12 and 17 years old and 16–65 years old living on the Balearic Islands, which has been described in more detail elsewhere [26,27]. Participants were invited to provide blood samples in order to determine the biochemical parameters. Five hundred and ninety-eight subjects (219 males and 379 females) took part in the biochemical phase. The reasons for not taking part were (a) the subjects declined or (b) the parents of adolescents did not authorize the blood sample provision. Participants were divided into two groups: adolescents (12–17 years; 146 males and 218 females) and adults (18–65 years; 73 males and 161 females). The study protocol was in accordance with the Declaration of Helsinki and was approved by the Balearic Islands Ethics Committee (Approval Reference Number IB/2251/14-PI).

### 2.2. General Questionnaire 

Dietary questionnaires and a global questionnaire incorporating questions related to socio-economic status (based on the occupation and classified as low, medium and high, according to the Spanish Society of Epidemiology methodology [28]), education level (grouped according to years and type of education: low, <6 years at school; medium, 6–12 years of education; high, >12 years of education) and lifestyle factors and health status were utilized (i.e., smoking habit (no; yes; occasionally, <1 cigarette/day) and alcohol consumption (no; yes)).

Dietary questionnaires were interviewer administered and included two 24-h diet recalls and a semiquantitative food-frequency questionnaire (FFQ) previously validated [29] and covers 145 food items (118 of the original validated FFQ plus the most characteristic Balearic Islands foods in order to make the interviewee answer easy) arranged by food type. Frequency of food consumption was based on times that food items were consumed (frequency per day, week or month). Consumption <1/month was considered no consumption. Daily consumption (g) was determined by dividing the reported amount of the intake by the frequency per day (d). The 24-h recall was carried out twice during the study period; the first in the warm season (May–September) and the second in the cold season (November–March). This was to avoid the influence of seasonal variations. To avoid bias brought on by day-to-day intake variability, the questionnaires were administered from Monday–Sunday. Conversion of food into nutrients was made using a self-made computerized program based on Spanish [30,31] and European [32] food composition tables and complemented with food composition data available for Majorcan food items [33]. Volumes and portion sizes were reported in natural units, household measures or with the aid of a book of photographs [34]. Information about food consumption patterns was obtained from the food frequency questionnaire, whereas information on nutrient intake was derived from the average food daily consumption reported in the two 24-h recalls [33]. Identification of underreported food intake was made using the energy intake:basal metabolic rate (EI:BMR) ratio. In adults, an EI:BMR ratio <1.14 classified the individual as an under-reporter [35]. In adolescents, an EI:BMR ratio of <0.92 (boys) and <0.85 (girls) was considered to represent underreporting [36]. Under-reporters did not take part in the biochemical phase in order to avoid a confounding factor.

### 2.3. Mediterranean Dietary Pattern

The MDP was defined according to a previously-defined score indicating the degree of adherence to the traditional MD [1,8]. This Mediterranean dietary score (MDS) was converted to relative percentage of adherence using a previously described method [37] that will now be briefly summarized.

An energy-adjusted value was obtained for each individual for the daily consumption of legumes, cereals and roots (including bread and potatoes), fruit (including nuts), vegetables, fish, meat (and meat products) and milk (and milk products). The alcohol consumption in adolescents must be null, and values above the reference indicate consumption of alcohol on the part of adolescents. In adults, in order to score ‘moderate alcohol consumption’, a transformation centered at the level of consuming 30 g/day for men (30–(30–absolute alcohol intake)) and 20 g/day for women (20–(20–absolute alcohol intake)) was used to obtain the highest value for men consuming 30 g/day or women consuming 20 g/day and progressive lower values as the consumption was lower or higher than these values. Information about the consumption of all these food items was obtained from the food-frequency questionnaire. The daily intake of fatty acids was calculated as the average intake recorded from the two 24-h recalls, and the monounsaturated fatty acids (MUFA):saturated fatty acids (SFA) ratio was calculated.

All these values were standardized as a *Z* value [31,34]. A *Z* score expresses the difference between the individual’s measurement and the mean value of the reference population (in this case, the study population), as a proportion of the standard deviation (SD) of the reference population (observed intake − mean intake/SD). In adults, the total MDS was computed by adding up all the *Z* scores obtained for the favorable or more Mediterranean’ dietary components (legumes, cereals and roots, fruit, vegetables, fish, moderate alcohol, MUFA:SFA ratio) and subtracting the *Z* value obtained from the consumption of meat and milk:

∑*Z*_i_ = *Z*_legumes_ + *Z*_cereals and roots_ +*Z*_fruit_ + *Z*_vegetables_ + *Z*_fish_ + *Z*_moderate alcohol_ + *Z*_MUFA:SFA_ − *Z*_meat_ − *Z*_milk_

In adolescents, the total MDS was computed by adding up all the *Z* scores obtained for the favorable or ‘more Mediterranean’ dietary components (legumes, cereals and roots, fruit, vegetables, fish and MUFA:SFA ratio) and subtracting the *Z* value obtained from the consumption of meat, whole milk (mainly high in fat) and alcohol:

∑*Z*_i_ = *Z*_legumes_ + *Z*_cereals and roots_ + *Z*_fruit_ + *Z*_vegetables_ + *Z*_fish_ + *Z*_MUFA:SFA_ − *Z*_meat_ − *Z*_whole milk_ − *Z*_alcohol_

The MDS was converted to the relative percentage of adherence using the range of values of the sample. This percentage ranged from 100 (maximum adherence) to 0 (minimum adherence):

Adherence (Percentage_i_) = (∑*Z*_i_ − ∑*Z*_min_) × 100/(∑*Z*_max_ − ∑*Z*_min_)

Once the percentage of adherence to the MDP was calculated, the variables that could determine a higher or lower adherence were assessed.

### 2.4. Anthropometric Measurements 

Height was determined using a mobile stadiometer (Kawe 44444, Kirchner & Wilhelm GmBH Co., KG, Asperg, Germany), with the subject’s head in the Frankfurt plane. Body weight was determined to the nearest 100 g using a digital scale (Tefal, sc9210, Groupe SEB, Rumilly, France). The subjects were weighed in bare feet and light underwear, which was accounted for by subtracting 300 g from the measured weight. The body mass index (BMI) was also calculated.

Triceps skinfold (TSF) and subscapular skinfold thickness (SCSF) were measured using a Holtain skinfold caliper (Tanner/Whitehouse, Crymych, U.K.), and the mean of three measurements (right arm) was used to calculate body fat (BF) as described previously [34].

Waist circumference (WC) and hip circumference (HC) were measured using a non-stretchable measuring tape (Kawe 43972, Kirchner & Wilhelm GmBH Co., KG, Asperg, Germany). The subjects were asked to stand erect on a flat surface in a relaxed position with both feet together. WC was measured as the smallest horizontal girth between the costal margins and the iliac crests at minimal respiration. HC was taken as the greatest circumference at the level of the greater trochanter (the widest portion of the hip) on both sides. For both WC and HC, two measurements were made to the nearest 0.1 cm, and the mean of the two readings was taken as the final value. The waist-to-hip ratio (WHR) was also calculated.

Automated (Omron, M4-I, Healthcare Co. Ltd., Kyoto, Japan) blood pressure (BP) measurements to the nearest 1 mmHg were taken from seated participants with the right arm resting and palm facing upward. Two readings were taken 5 min apart, and the mean of the two readings was taken. If the difference between the first and the second reading was ≥10 mmHg for systolic blood pressure (SBP) and/or ≥6 mmHg for diastolic blood pressure (DBP), then a third measurement was made, and the mean of all three measurements was taken.

### 2.5. Metabolic Syndrome Definition

The program’s Adult Treatment Panel III [38,39] was used to define MetS in subjects aged 18–65 years. Participants were defined as having MetS if they met or exceeded the criteria for three or more of the following five variables: (1) waist circumference >102 cm in men and >88 cm in women; (2) serum triglyceride ≥150 mg/dL; (3) HDL-cholesterol <40 mg/dL in men and <50 mg/dL in women; (4) BP ≥130/85 mmHg; and (5) fasting plasma glucose level ≥100 mg/dL.

The MetS definition used in adolescents (12–17 years) was that for adolescents proposed by de Ferranti et al. [40], and their criteria were based closely on The Adult Treatment Panel III (ATP III) [39]. Participants were defined as having MetS if they met or exceeded the criteria for three or more of the following five variables: triglyceridemia (TG) ≥1.1 mmol/L; high-density lipoprotein cholesterol (HDL-c) <1.3 mmol/L (boys aged 15–17 years <1.17 mmol/L); fasting blood glycaemia ≥6.1 mmol/L; waist circumference (cm) >75th percentile for age and sex [41]; SBP and/or DBP (mm Hg) >90th percentile for age, sex and height [42].

### 2.6. Biochemical Measurements

Venous blood samples were obtained from the antecubital vein in suitable vacutainers at 08:00 h of the interview day after 12-h overnight fasting conditions. Plasma was obtained after centrifugation of the blood samples at 900× *g* for 20 min at 4 °C. The plasma phase was then collected and freshly used for biochemical assays. Circulating levels of adiponectin (AD), leptin, tumor necrosis factor alpha (TNF-α) and plasminogen activator inhibitor 1 (PAI-1) were determinated by the fluorokine MultiAnalyte Profiling (MAP) Human Obesity Base kit based on Luminex^®^-technology (R&D systems^®^, Minneapolis, MN, USA). High-sensitivity C-reactive protein (hs-CPR) was measured by nephelometry (Dade Behring BNII, Marburg, Germany).

### 2.7. Statistical Analysis

Statistical analyses were performed with SPSS Version 24.0 for Windows (SPSS Inc., Chicago, IL, USA). According to previous studies in which sex differences in inflammatory markers were found [43,44], in the present study, all tests were stratified by sex and age. The median value of adherence to the MD was calculated in each age group, and subjects were categorized into two groups (above and below the median value). All continuous variables were expressed as the mean ± standard deviation. Anthropometric measurements were compared between both age groups in each sex. Inflammatory markers were compared between groups of adherence to the MD (above and below the median) in each sex and age group. Differences between group means were tested using Analysis of Variance (ANOVA). Differences in MetS prevalence were calculated by means of χ^2^. Correlation between inflammatory markers and dietary components of the MDS were also assessed using the Pearson correlation coefficient. A *p*-value ≤ 0.05 was considered statistically significant.

## 3. Results 

Table 1 shows the general characteristics of male and female subjects according to age group. In both the male and female subjects, there were significant differences in weight, height, BMI, TSF, WC, HC, WHR, total body fat and MetS. All these parameters were significantly higher in the 18–65 group vs. the 12–17 group.

**Figure 1:**
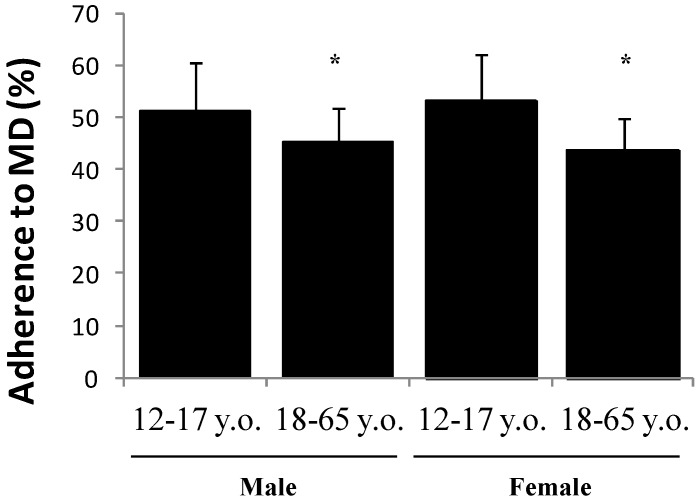
Adherence to Mediterranean dietary (MD) pattern expressed as a percentage (%) ± standard deviation (SD) in male and female subjects studied. y.o.: years old. Statistically-significant differences: * *p* < 0.05.

Figure 1 represents the percentage of adherence to the MDP of male and female subjects according to age group. In both sexes, the adherence to the MDP is significantly lower in the older groups. The adherence to the MD was significantly different between male adolescents (51.3 ± 9.0) and adults (45.1 ± 6.8) and between female adolescents 53.1 ± 9.1 and adults 43.7 ± 6.0. On the contrary, no significant differences are evidenced when comparing the adherence between the two sexes.

Table 2 shows levels of inflammatory markers in adult male subjects according to the adherence to the MDP. Regarding adiponectin levels, statistically-significant differences were obtained in adult subjects, for which higher levels of adiponectin were associated with higher adherence to the MDP. The other biomarkers (leptin, TNF-α, PAI-1 and hs-CRP) values were statistically significant in adult male subjects, reporting plasma lower levels among subjects with higher adherence to the MDP.

Table 3 shows levels of inflammatory markers in females in relation to adherence to the MDP. Adiponectin and TNF-α level did not show any statistically-significant difference depending on the adherence to the MDP. Leptin values were statistically significant for adolescents, for which lower levels of leptin were associated with higher adherence. PAI-1 values were statistically significant for adults, for which a lower level had higher adherence. Hs-CRP values were statistically significant for both groups, evidencing lower levels of the subjects with higher adherence. No significant correlations were evidenced between the different individual food groups (fruits, vegetables, etc.) and the inflammatory biomarkers (data not shown).

## 4. Discussion

The main finding of the present study is that low adherence to the MDP was directly associated with a worse profile of plasmatic inflammation markers, especially in the male adult population, whereas the female population only showed significance for PAI-1 and hs-CRP. The present results also indicate that with increasing age, there is an increase in weight, BMI, TSF, WC, HC, WHR, total body fat and MetS (%), but a decrease in the adherence to MDP in both sexes. The adherence to the MDP (51–53% in adolescents and 43–45% in adults) is similar to that observed in the population of the Balearic Islands, both in the adult population [45] and in the adolescent population [46]. Compared to adults, adolescents had higher adherence to the MDP, however, it cannot be discarded that adolescents underreported their sweets and snacks consumption.

Weight gain is a common physiological process associated with aging derived from a decrease in BMR in both men and women, especially, in the case of women, during the transition to menopause derived from hormonal changes [47,48]. Aging also induces important changes in body fat distribution with an increased tendency for central or visceral fat distribution (android pattern). Obesity and increased central body fat are directly related to metabolic alterations and an increased risk of cardiovascular disease. In addition, older people are more prone to low-grade chronic inflammation, which can contribute to worsen chronic diseases [49]. In the Balearic Islands, as well as in other Mediterranean countries, the prevalence of MetS increases progressively with age, and males were also at higher risk for MetS [50,51]. It has been also reported that subjects with lower MDP adherence exhibited higher occurrence of MetS and all its components [52].

Regarding markers of inflammation, higher adherence to the MPD was associated with higher levels of adiponectin and lower levels of leptin, TNF-α, PAI-1 and hs-CRP in adults, but not in young subjects. In females, higher adherence was associated with lower levels of leptin in the young group, PAI-1 in adults and hs-CRP in both groups. Adiponectin is an adipokine mostly secreted from adipose tissue with anti-diabetic, anti-obesity and anti-inflammatory effects [53]. The intake of fish, omega-3 supplementation and adherence to an MDP seem to be associated with an increase in adiponectin levels [54,55]. However, in the present study, a lack of a statistically-significant correlation between fish or any other food groups was found, suggesting cumulative effects of all MD components, and not a clear effect of specific diet component. In type 2 diabetic subjects, an intervention with an MD had a total follow-up of 8.1 years [56]. Accordingly, the low adiponectin levels evidenced in males with an adherence to MDP under the median value could contribute to obesity and cardiovascular risk since this group presents a higher prevalence of MetS. Similar results were obtained in a previous study that showed a decrease in adiponectin concentration in response to the MD, but this decrease only reached statistical significance in men [57]. Leptin is another adipokine synthesized by adipocytes proportionally to adipocyte volume and total fat mass and also plays a key role in the regulation of appetite [58]. Diverse intervention studies reported that a hypocaloric diet based on an MDP resulted in a notable reduction in plasma concentrations of inflammatory biomarkers including leptin [59,60,61].

However, these changes in adipokines are only evident when they were associated with weight loss. MD with weight loss significantly reduced plasma leptin levels and increased plasma adiponectin levels [60,61]. PAI-1, mainly produced by endothelial cells, has been positively associated with visceral adiposity and has been shown to be risk factor for thrombotic diseases since this factor is the main inhibitor of fibrinolysis. The higher levels reported in PAI-1 in both adult males and females with a low adherence to MDP suggest a higher risk for thrombotic diseases. Several studies evidenced that modifying the diet towards MD leads to a decrease in PAI-1 [60,62,63].

In the present study, adult males with higher adherence to the MD showed better inflammatory profile vs. the group with adherence under the median value. The changes are less prominent in adult females when comparing the degree of adherence to MD. It has been evidenced that estrogens are cardio-protective and also tend to reduce fat intake and increase energy expenditure, reducing fat accumulation, mainly visceral fat [64]. The visceral fat is directly associated with the production of pro-inflammatory mediators, and in females, the minor accumulation of this fat can minimize the differences in the measured markers [65]. In males, changes in the quality of the diet such as increasing the intake of more healthy vegetable oils could increase the degree of the observed change in the inflammatory markers.

There are studies showing that increasing the adherence to MD, weight reduction and exercise interventions are associated with decreases in markers of systemic inflammation, including IL-6, TNF-α and CRP [52,60,66,67,68]. In this sense, hs-CRP seems to be the most adequate biomarker of metabolic syndrome-linked inflammation since it showed higher plasma concentrations in the groups with lower adherence to the MDP. The results are in accordance with previous reports reporting a significant reduction of hs-CRP levels in subjects who implemented an MDP, independently of weight loss [24,50,60]. Accordingly, in a subcohort of the PREDIMED (Prevención con Dieta Mediterránea) study, a significant reduction of circulating CRP and IL-6 after 12 months of intervention with MD was evidenced [67]. It has been suggested that the inverse relation between hs-CRP with MD score could be associated with a higher unsaturated fat amount of these foods, which may predispose to abdominal obesity and lipidic oxidation [52].

Changes in the traditional MDP toward the modern Western diet are taking place, representing a dietary globalization. Nowadays, a diet rich in meat, processed foods and sweets is increasingly more common in the population, affecting young and adult subjects. However, a high adherence to the MDP is related to a lower risk of suffering pathologies such as cardiovascular diseases or cancer [3,4,5,6]. In this sense, the beneficial effect of the MDP against CVD has been mainly attributed to its effects controlling classical atherosclerosis risk factors. Lately, it has been suggested that an anti-inflammatory effect in the vascular wall may be another important mechanism to explain the link between the MDP and low cardiovascular mortality [9]. The important role of inflammation in the pathogenesis of atherosclerosis has led to the belief that dietary preventive measures act in part by modifying related inflammatory pathways [69]. Indeed, atherosclerosis has long been considered the result of lipid accumulation in the artery wall, but there is currently compelling evidence that inflammation plays a key role at all stages of the disease [70]. Early phases of atherosclerosis involve the recruitment of inflammatory cells from the circulation, their adhesion to endothelium and finally migration to subendothelial space, a complex process mediated by inflammatory stimuli, which involves cytokine production and upregulation of adhesion molecules on endothelial cells and leucocytes [71]. There are studies in Mediterranean populations suggesting that the MDP has anti-inflammatory effects [24,72], which was also ascertained in the U.S. population [73]. Furthermore, other research suggests that a long-term consumption of a Mediterranean-style diet may be an effective dietary strategy for protecting against metabolic syndrome, a risk factor for type 2 diabetes mellitus and CVD [74]. Finally, recent studies evidenced that the quality of the Mediterranean diet modifies molecular parameters such as the methylation profile and also the expression of microRNAs in white blood cells, which are related to the modulation of inflammation [75,76]. Furthermore, a reduction of pro-inflammatory mediators by adipocytes could reduce immune cell infiltration in adipose tissue and, hence, contribute to the reduction of the low-grade inflammation [65].

## 5. Strengths and Limitations

This study provides further information on the relationship between the MDP and inflammatory markers. However, this study has several limitations. First, the present cross-sectional design gives limited ability to elucidate the causal relationship between inflammatory markers and MDP. Second, there is a potential lack of generalization of the results due to the sample size obtained for each age group of males and females. Third, despite the guidance of licensed dieticians, diet was self-reported, and we cannot rule out some recall bias.

## 6. Conclusions

The present results show that low adherence to the MDP is directly associated with a worse profile of plasmatic inflammation markers, especially in the male adult population, with the female population only showing significance for PAI-1 and hs-CRP. Evidence that overeating and adiposity contribute to systemic inflammation and development of metabolic syndrome raises the possibility that lifestyle interventions may provide effective means of reducing risk factors for CVD. Future longitudinal studies can explore whether the decrease in risk associated with lifestyle changes is mediated by decreases in systemic inflammation.

## Figures and Tables

**Table 1:** General characteristics of male and female subjects studied.

	Age (Years)	
**Male (*n* = 219)**	**12–17 (*n* = 146)**	**18–65 (*n* = 73)**
Weight (kg), mean ± SD	62.7 ± 12.6	82.2 ± 14.7 *
Height (cm), mean ± SD	170.5 ± 9.4	174.9 ± 6.4 *
BMI (kg/m^2^), mean ± SD	21.4 ± 3.1	26.9 ± 5.1 *
TSF (mm) ± SD	10.1 ± 4.0	16.3 ± 7.1 *
WC (cm) ± SD	72.2 ± 7.8	89.6 ± 13.3 *
HC (cm) ± SD	91.5 ± 9.0	103.6 ± 8.2 *
WHR ± SD	0.79 ± 0.05	0.86 ± 0.06 *
Total body fat (%)	12.9 ± 6.0	20.6 ± 7.1 *
MetS (%)	8.6	16.3 *
**Female (*n* = 379)**	**(*n* = 218)**	**(*n* = 161)**
Weight (kg), mean ± SD	57.3 ± 9.6	64.3 ± 13.1 *
Height (cm), mean ± SD	161.4 ± 6.2	162.8 ± 11.2 *
BMI (kg/m^2^), mean ± SD	21.9 ± 3.3	26.7 ± 5.0 *
TSF (mm) ± SD	14.3 ± 4.4	23.7 ± 7.3 *
WC (cm) ± SD	67.5 ± 6.5	76.9 ± 11.1 *
HC (cm) ± SD	94.7 ± 8.0	102.5 ± 9.4 *
WHR ± SD	0.71 ± 0.06	0.75 ± 0.05 *
Total body fat (%)	15.2 ± 5.8	28.9 ± 6.2 *
MetS (%)	3.4	7.2 *

Abbreviations: SD: standard deviation; BMI: body mass index; TSF: triceps skinfold; WC: waist circumference; HC: hip circumference; WHR: waist-to-hip ratio; MetS: metabolic syndrome. Statistically significant differences: * *p* < 0.05.

**Table 2:** Levels of inflammatory markers in adult male subjects according to the adherence to the MDP.

Adherence to MDP (%)	*n*	Adiponectin (µg/mL)	Leptin (ng/mL)	TNF-α (pg/mL)	PAI-1 (ng/mL)	hs-CRP (mg/mL)
12–17 years old						
Above median value (≥50%)	68	13.8 ± 5.8	10.4 ± 8.6	5.4 ± 4.7	577 ± 212	0.16 ± 0.26
Under median value (<50%)	78	14.4 ± 5.8	7.6 ± 9.1	6.0 ± 5.1	563 ± 229	0.19 ± 0.38
18–65 years old						
Above median value (≥50%)	40	13.1 ± 6.7	9.4 ± 7.3	7.9 ± 2.4	201 ± 29	0.17 ± 0.18
Under median value (<50%)	33	9.5 ± 2.4 *	16.0 ± 9.5 *	12.3 ± 3.0 *	262 ± 32 *	0.41 ± 0.42 *

Values are expressed as mean ± standard deviation. Abbreviations: MDP: Mediterranean dietary pattern; TNF-α: tumor necrosis factor alpha; PAI-1: plasminogen activator inhibitor 1; hs-CRP: high-sensitivity C-reactive protein. Statistically significant differences between male by Analysis of Variance (ANOVA): * *p* < 0.05.

**Table 3:** Levels of inflammatory markers in adult female subjects according to the adherence to the MDP.

Adherence to MDP (%)	*n*	Adiponectin (µg/mL)	Leptin (ng/mL)	TNF-α (pg/mL)	PAI-1 (ng/mL)	hs-CRP (mg/mL)
12–17 years old						
Above median value (≥50%)	90	16.4 ± 6.5	24.7 ± 11.0	5.4 ± 4.5	476 ± 236	0.07 ± 0.08
Under median value (<50%)	128	15.0 ± 6.0	36.3 ± 13.9 *	5.3 ± 4.8	538 ± 266	0.16 ± 0.13 *
18–65 years old						
Above median value (≥50%)	78	10.3 ± 1.5	44.3 ± 25.1	7.3 ± 3.1	204 ± 32	0.18 ± 0.23
Under median value (<50%)	83	11.1 ± 1.1	43.0 ± 24.0	7.7 ± 2.7	298 ± 43 *	0.28 ± 0.32 *

Values are expressed as mean ± standard deviation. Abbreviations: MDP: Mediterranean dietary pattern; TNF-α: tumor necrosis factor alpha; PAI-1: plasminogen activator inhibitor 1; hs-CRP: high-sensitivity C-reactive protein. Statistically significant differences between female by ANOVA: * *p* < 0.05.
